# Knowledge, perception, and attitudes of medical students towards antimicrobial resistance and stewardship: an observational cross-sectional study from Palestine

**DOI:** 10.1186/s12909-024-05276-7

**Published:** 2024-03-18

**Authors:** Mohammad Abuawad, Azza Ziyadeh-Isleem, Aya Mahamid, Saja Quzmar, Enas Ammar, Ramzi Shawahna

**Affiliations:** 1 Department of Biomedical Sciences, Faculty of Medicine and Health Sciences, An-Najah National University, Nablus, Palestine; 2 Department of Medicine, Faculty of Medicine and Health Sciences, An-Najah National University, Nablus, Palestine; 3 Clinical Research Center, An-Najah National University Hospital, Nablus, 44839 Palestine

## Abstract

**Background:**

Antimicrobial resistance is a global health concern that contributes to significant mortality and morbidity. This study investigated knowledge, perceptions, and attitudes of medical students in Palestinian universities towards antimicrobial resistance and stewardship. The study also investigated associations between variables of students with their knowledge, perceptions, and attitudes.

**Methods:**

A questionnaire was used in this cross-sectional study. Medical students in Palestinian universities were surveyed in this study. In addition to the demographic variables of the medical students, the questionnaire measured knowledge, perceptions, and attitudes of medical students in Palestinian universities towards antimicrobial resistance and stewardship.

**Results:**

In this study, 384 medical students returned complete questionnaires. Of the medical students, 269 (70.1%) were female, 215 (56.0%) lived in urban areas, and 244 (63.5%) were in their clinical training years. Medical students in the clinical phase demonstrated higher knowledge about microbial resistance compared to preclinical students as evidenced by a mean score of 89.7 ± 15.9 compared to 74.0 ± 26.4, respectively (*p* < 0.05). Similarly, clinical students displayed higher score regarding antimicrobial resistance perception, with a mean score of 85.7 ± 15.6, contrasted with 72.6 ± 24.1 among preclinical students. In terms of knowledge pertaining to antimicrobial stewardship, clinical students scored higher with a mean of 63.4 ± 28.5 compared to 54.5 ± 31.5 among preclinical students. Regarding attitudes, clinical students also demonstrated a higher mean score of 67.6 ± 22.6 in contrast to 61.1 ± 24.6 among preclinical students.

**Conclusion:**

The medical students are the future workforce of physicians in any healthcare system. Therefore, increasing knowledge of the medical students about and how to combat antimicrobial resistance can help save lives and improve patient outcomes. More studies are needed to find the best ways to increase knowledge, perceptions, and attitudes of medical students towards antimicrobial resistance and antimicrobial stewardship.

## Introduction

Antimicrobial resistance is a natural biological phenomenon in which microorganisms that were previously susceptible to a particular antimicrobial agent become no longer susceptible [1]. This process can occur naturally over time, often as a result of genetic changes. However, the overuse and misuse of antimicrobial agents can accelerate the development of antimicrobial resistance [2]. This can lead to the emergence of pathogens that are resistant to commonly used antimicrobial agent, making it difficult to treat common infectious diseases.

The World Health Organization reported that antimicrobial resistance can make it challenging and more costly to treat a range of common infections, which can result in delays in providing effective treatment or, in the worst-case scenarios, inability to provide suppress the microbes [3]. Many medical advances in recent years, including chemotherapy for cancer treatment and organ transplantation, rely on the availability of effective antimicrobial agents. The inevitable outcome of resistance is an increased incidence of illness, longer recovery times, a greater risk of complications, and higher mortality rates [4]. Therefore, addressing antimicrobial resistance in developing countries is an urgent matter. Because of the ineffectiveness of some antimicrobial agents to which specific pathogens have developed resistance, there is a significant demand for broad-spectrum agents, which imposes an economic burden on developing countries [5].

Different health organizations have published policies to promote rationale use reduce misused of antimicrobial agents. Previous studies have shown that the increased use of antimicrobial agents was associated with the emergence of antimicrobial resistance worldwide [1, 2, 4]. Additionally, studies have shown that antimicrobial agents were inappropriately prescribed by infections that are known to be caused by viruses including common cold. According to some estimates, up to 55% of the antimicrobial agents there were prescribed to treat upper respiratory infections were deemed unnecessary [6, 7]. It has been argued that policymakers should coordinate efforts to improve provision of healthcare and reduce antimicrobial resistance.

The World Health Organization stated that providing education to healthcare professionals and medical students on appropriate antimicrobial prescribing practices or “antimicrobial stewardship” is a crucial component of all efforts aimed at containing antimicrobial resistance [8]. It has emphasized the significance of providing sufficient and efficient training to medical students to promote the judicious use of antibiotics. In order to deal with the issue of antimicrobial resistance and to assist prescribers in their endeavors to treat patients effectively, antimicrobial stewardship programs have been initiated around the world.

Education on antimicrobial resistance and stewardship is crucial in medical schools to equip future physicians with the necessary knowledge. Research suggests that starting antimicrobial stewardship programs early in medical training is important [9–11]. Previous studies have demonstrated that the attitudes and behaviors of healthcare professionals regarding antibiotic usage can be notably shaped by education during undergraduate training [12, 13]. Therefore, this cross-sectional study was conducted to determine medical students’ views on antimicrobial resistance and antimicrobial stewardship. The study aimed to investigate the level of knowledge, perceptions, and attitudes towards antimicrobial resistance and stewardship among medical students in Palestinian universities.

## Methodology

### Study design and settings

A questionnaire was used in this cross-sectional study. The questionnaires were distributed to the medical students between December 2022 and February 2023. By doing so, we hoped to gain valuable insights into medical students’ understanding of antimicrobial resistance and stewardship, which could aid in the development of effective educational interventions to improve appropriate antimicrobial use and combat the rising threat of antimicrobial resistance.

### Study population, inclusion and exclusion criteria

The study population was medical students in the Palestinian universities including An-Najah National University and Al-Quds University. The medical students were invited to participate in the study regardless of their sex, age, academic year, or training stage. In Palestine, medical education spans six years, divided into two phases. The first phase, encompassing the initial three years, focuses on preclinical studies where students delve into fundamental aspects of medicine such as anatomy, physiology, medical microbiology, pathology, and related subjects. The second phase, comprising the subsequent three years, is dedicated to clinical practice and education, during which students engage in hands-on learning in hospitals, specializing in various medical disciplines including medicine, surgery, and practical care. Before participation the students were asked to provide and informed consent. Those who refused to provide informed consent and those who did not complete all items in the questionnaire were excluded. The inclusion criteria encompassed Palestinian undergraduate medical students at both preclinical and clinical stages, with the exception of first-year students. Specifically, participants were enrolled at An-Najah National University and Al-Quds University. Those who did not meet these criteria or were pursuing disciplines outside of medicine were excluded from the study. 

### Sample size and sampling techniques

The largest sample size was calculated based on the largest number of medical students in the Palestinian universities. The sample was calculated based on a population of about 6,000 medical students in all academic years in the different Palestinian universities. Using a 95% confidence interval, a response distribution of 50%, and accepting a margin of error of 5%, the sample size was 362 medical students. A paper designed questionnaire was used to gather data from medical students in both Pre-clinical and Clinical stages via face-to-face interviews, as this method was deemed the most convenient for data collection. A convenience sampling technique was used to recruit the medical students from the different universities.

### Variable definitions

The variables were as follows:

- The dependent variables were knowledge and perceptions regarding antimicrobial stewardship and antimicrobial resistance. These variables were expressed in summed numbers and percentages as determined from the items in the study tool.
- The independent variables were the characteristics of the medical students like: sex, marital status, place of residence (urban meant living in cities and rural meant living in villages), and academic year. Age was collected as a continuous variable in years, sex was binary as either male or female, the marital status was also binary as single or married, and the place of residence was either rural or urban.

### Study tool, validity and reliability

The study tool used was adapted from a previous study [14]. The tool was tested for validity and reliability. The items related to knowledge, perceptions, and attitudes toward microbial resistance and antimicrobial stewardship were shown to be reliable and internally consistent.

### Bias

The sample was diversified, the students were recruited from more than one university, and the tool was previously used in other studies. However, The study utilized multiple-choice questions with options of ‘yes,’ ‘no,’ or ‘I don’t know.’ This format potentially allowed students to make guesses when answering the questions.

### Statistical analysis

The data were entered and analyzed using IBM SPSS v.21.0. Scores were calculated as percentages of correct answers divided by the number of items in each domain. The data were tested for normality using Shapiro-Wilk test. The normality of distribution was assessed using Kolmogorov-Smirnov test. As the data were normally distributed, means and standard deviations (SD) were used. Differences between the groups were tested using Student’s t-tests. A *p*-value of < 0.05 was considered as statistically significant.

## Results

### Characteristics of the medical students

A total of 384 medical students completed the study. Of the medical students, 269 (70.1%) were female, 215 (56.0%) lived in urban areas, and 244 (63.5%) were in their clinical training years. The detailed demographic and academic details of the medical students are shown in Table 1.

**Table 1:** Characteristics of the medical students

Variable	n	%
**Sex**		
Male	115	29.9
Female	269	70.1
**Place of residence**		
Urban	215	56.0
Rural	169	44.0
**Academic year**		
Basic medical training	140	36.5
Clinical training	244	63.5

### Knowledge of medical students about microbial resistance

The medical students demonstrated high awareness/knowledge about microbial resistance as evident by the number and percentage of correct answers on the knowledge items. The detailed answers of the medical students on the knowledge items about microbial resistance are shown in Table 2.

**Table 2:** Knowledge of medical students about microbial resistance

		No		Yes		I am not sure	
#	**Item**	**n**	**%**	**n**	**%**	**n**	**%**
1	I have heard about the term “antimicrobial resistance”	8	2.1	374	97.4	2	0.5
2	Poorly designed dosing regimens can contribute to antimicrobial resistance	31	8.1	327	85.2	26	6.8
3	The use of broad-spectrum antibiotics promotes antimicrobial resistance	35	9.1	333	86.7	16	4.2
4	Antibiotics can kill viruses	315	82.0	38	9.9	31	8.1
5	Bacteria cause flu and common cold	263	68.5	77	20.1	44	11.5

### Association between variables of the students and knowledge about antimicrobial resistance

The overall mean knowledge score was 84.0% ± 21.7%. When the knowledge scores of the medical students about antimicrobial resistance were compared between groups, those who were in their clinical training years demonstrated significantly higher knowledge that those who were in their basic training years. Sex, marital status, and place of residence were not statistically associated. The details of these associations are shown in Table 3.

**Table 3:** Association between variables of the students and knowledge about antimicrobial resistance

			Knowledge about antimicrobial resistance		
Variable	n	%	Mean	SD	*p*-value
**Sex**					
Male	115	29.9	84.9	21.0	0.590
Female	269	70.1	83.6	22.0	
**Marital status**					
Single	372	96.9	83.8	21.8	0.327
Married	12	3.1	90.0	16.0	
**Place of residence**					
Urban	215	56.0	85.4	21.1	0.143
Rural	169	44.0	82.1	22.3	
**Academic year**					
Basic medical training	140	36.5	74.0	26.4	< 0.001
Clinical training	244	63.5	89.7	15.9	

### Perceptions of antimicrobial resistance

Similar to knowledge, the medical students expressed high perception of antimicrobial resistance as evident by the number of correct answers on the perception items. The detailed answers of the medical students on the perception of antimicrobial resistance are shown in Table 4.

**Table 4:** Perceptions of antimicrobial resistance

		No		Yes		I am not sure	
#	**Item**	**n**	**%**	**n**	**%**	**n**	**%**
1	Irrational use of antibiotics can harm the patient	8	2.1	355	92.4	21	5.5
2	Casual/common use of antibiotics in Palestine is appropriate	206	53.6	68	17.7	110	28.6
3	Broad-spectrum antibacterial are used unnecessarily when narrow-spectrum antibiotics are available	41	10.7	292	76.0	51	13.3
4	Poor patient adherence to prescribed antibiotics can be a cause of antimicrobial resistance	25	6.5	324	84.4	35	9.1
5	It is important to follow the appropriate duration of antimicrobials to prevent the development of resistance	9	2.3	363	94.5	12	3.1
6	Antibiotic use should be reduced	20	5.2	325	84.6	39	10.2

### Association between variables of the students and their perceptions of antimicrobial resistance

The overall mean perception score was 80.9% ± 20.1%. Again, similar to knowledge, perception scores were significantly higher for the medical students who were in their clinical training years compared to those who were in their basic training years as shown in Table 5. The other variables were not statistically significant.

**Table 5:** Association between variables of the students and their perceptions of antimicrobial resistance

			Perception of antimicrobial resistance		
Variable	n	%	Mean	SD	*p*-value
**Sex**					
Male	115	29.9	81.4	19.5	0.749
Female	269	70.1	80.7	20.4	
**Marital status**					
Single	372	96.9	80.8	20.3	0.367
Married	12	3.1	86.1	12.0	
**Place of residence**					
Urban	215	56.0	81.2	20.9	0.747
Rural	169	44.0	80.6	19.2	
**Academic year**					
Basic medical training	140	36.5	72.6	24.1	< 0.001
Clinical training	244	63.5	85.7	15.6	

### Knowledge about antimicrobial stewardship

Of the medical students, the majority (66.1%) were not confident about their knowledge about antimicrobial stewardship. On the other hand, the majority (81.8%) believed that antimicrobial stewardship aims to optimize antimicrobial use. The detailed answers of the medical students are shown in Table 6.

**Table 6:** Knowledge about antimicrobial stewardship

		No		Yes		I am not sure	
#	**Item**	**n**	**%**	**n**	**%**	**n**	**%**
1	I have adequate knowledge about antimicrobial stewardship	254	66.1	108	28.1	22	5.7
2	Antimicrobial stewardship aims to optimize antimicrobial use	15	3.9	314	81.8	55	14.3
3	Antimicrobial stewardship is the key component of a multifaceted approach for preventing the emergence of antimicrobial resistance	13	3.4	271	70.6	100	26.0

### Association between variables of the students and their knowledge about antimicrobial stewardship

The overall mean knowledge about antimicrobial stewardship score was 60.2% ± 29.9%. In this study, the knowledge about antimicrobial stewardship scores were significantly higher for the medical students who were married and in their clinical academic training years compared to those who were single and were in their basic medical training. Details of these associations are shown in Table 7.

**Table 7:** Association between variables of the students and their knowledge about antimicrobial stewardship

			Knowledge about antimicrobial stewardship		
Variable	n	%	Mean	SD	*p*-value
**Sex**					
Male	115	29.9	57.4	29.8	0.237
Female	269	70.1	61.3	29.9	
**Marital status**					
Single	372	96.9	59.5	29.9	0.016
Married	12	3.1	80.6	22.3	
**Place of residence**					
Urban	215	56.0	59.7	28.6	0.731
Rural	169	44.0	60.7	31.6	
**Academic year**					
Basic medical training	140	36.5	54.5	31.5	0.005
Clinical training	244	63.5	63.4	28.5	

### Perceptions of antimicrobial stewardship

The medical students expressed high perceptions of antimicrobial stewardship as evident by the correct answers on the perception items as shown in Table 8.

**Table 8:** Perceptions of antimicrobial stewardship

		No		Yes		I am not sure	
#	**Item**	**n**	**%**	**n**	**%**	**n**	**%**
1	Strong knowledge and awareness about correct antimicrobial use is important for better patient care	6	1.6	370	96.4	8	2.1
2	Pharmacists can play a role in awareness of correct antimicrobial usage	5	1.3	367	95.6	12	3.1
3	Antimicrobial stewardship should be incorporated into the healthcare system	2	0.5	360	93.8	22	5.7
4	Hospital pharmacist is an essential element of antimicrobial stewardship	10	2.6	332	86.5	42	10.9
5	Implementation of antimicrobial stewardship can ensure therapeutic efficacy of antibiotics and reduce antimicrobial resistance	9	2.3	318	82.8	57	14.8

### Association between variables of the students and their perceptions of antimicrobial stewardship

The overall mean knowledge about perceptions of antimicrobial stewardship score was 91.0% ± 17.6%. The perception scores were significantly higher for the medical students who were female and those who were in their clinical medical training compared to those who were male and were in their basic medical training as shown in Table 9.

**Table 9:** Association between variables of the students and their perceptions of antimicrobial stewardship

			Perception of antimicrobial stewardship		
Variable	n	%	Mean	SD	*p*-value
**Sex**					
Male	115	29.9	87.3	20.1	0.007
Female	269	70.1	92.6	16.2	
**Marital status**					
Single	372	96.9	90.8	17.8	0.142
Married	12	3.1	98.3	5.8	
**Place of residence**					
Urban	215	56.0	91.6	16.8	0.424
Rural	169	44.0	90.2	18.7	
**Academic year**					
Basic medical training	140	36.5	86.9	22.6	< 0.001
Clinical training	244	63.5	93.4	13.5	

### Attitudes towards learning about antimicrobial resistance and stewardship

In this study, the majority of the medical students expressed interest in learning about antimicrobial stewardship. On the other hand, only 36.7% believed that they have received adequate education about bacteria and the appropriate use of antibiotics during their university education. The detailed answers of the medical students are shown in Table 10.

**Table 10:** Attitudes towards learning about antimicrobial resistance and stewardship

		No		Yes		I am not sure	
#	**Item**	**n**	**%**	**n**	**%**	**n**	**%**
1	I read books or scientific articles about antibiotic resistance (book, article)?	127	33.1	226	58.9	31	8.1
2	I received adequate education about bacteria and the appropriate use of antibiotics during my university education	143	37.2	141	36.7	100	26.0
3	I would like to learn more about antibiotics and their resistance	33	8.6	306	79.7	45	11.7
4	I am interested in learning about antimicrobial stewardship	23	6.0	329	85.7	32	8.3

### Association between variables of the students and their attitudes towards learning about antimicrobial resistance and stewardship

The overall mean attitudes towards learning about antimicrobial resistance and stewardship score was 65.2% ± 23.5%. When the attitude scores were compared, the medical students who were male and those who were in their clinical training years had significantly higher scores that those who were female and were in their basic clinical training. The details of these associations are shown in Table 11.

**Table 11:** Association between variables of the students and their attitudes towards learning about antimicrobial resistance and stewardship

			Attitudes towards learning about antimicrobial resistance and stewardship		
Variable	n	%	Mean	SD	*p*-value
**Sex**					
Male	115	29.9	70.0	24.8	0.009
Female	269	70.1	63.2	22.7	
**Marital status**					
Single	372	96.9	65.6	23.6	0.098
Married	12	3.1	54.2	20.9	
**Place of residence**					
Urban	215	56.0	66.5	23.8	0.231
Rural	169	44.0	63.6	23.1	
**Academic year**					
Basic medical training	140	36.5	61.1	24.6	0.008
Clinical training	244	63.5	67.6	22.6	

## Discussion

The issue of antimicrobial resistance is a matter of global health and economics. To combat antimicrobial resistance, it is essential for healthcare providers to have a comprehensive understanding of the range of activity of antimicrobials and their relationship with resistance [14]. Countries with lower incomes, limited access to medications, and ineffective infection control measures may be particularly affected by this problem [15, 16]. Educating people about the proper use of antibiotics can help mitigate the issue of antibiotic resistance worldwide [14]. Despite healthcare systems offering instructional programs, there are still gaps in knowledge and inappropriate conduct. This study aims to evaluate what students in higher education know and think about antimicrobial resistance and stewardship.

In this study, more the majority of the medical students accurately recognized that antibiotic overuse and the inappropriate use of broad-spectrum antibiotics contribute to the promotion of antimicrobial resistance. The findings reported in this study were consistent with those reported from a previous study in Pakistan in which pharmaceutical, veterinary, and biology students acknowledged that the use of broad-spectrum antibiotics can result in antimicrobial resistance [14]. Similarly, the majority of the students believed that incorrect use of antibiotics can lead to antimicrobial resistance. A review of studies that were conducted to assess knowledge of medical students about antimicrobial resistance concluded that the medical students recognized that antimicrobial resistance was an issue but lacked basic knowledge about antimicrobial resistance [17]. These findings support the notion that antimicrobial resistance is caused by the misuse of antibiotics and poorly designed antibiotic treatment plans. Moreover, a recent extensive study involving healthcare workers, medical students, and adult communities in Arabic region showed unsatisfactory knowledge and perceptions regarding appropriate antibiotic usage [13]. Approximately 25% of respondents believed antibiotics could treat viral infections, and there was a misconception that antibiotics always remain effective for treating the same infection in the future [18]. Another large study conducted Arab populations involving 11 Arabic country reported poor knowledge of antibiotic usage and AMR [19].

The medical students who participated in this study reported poor knowledge about antimicrobial stewardship. When knowledge was compared between the groups, the medical students who were in their clinical training years had higher awareness and attitudes towards both antimicrobial resistance and antimicrobial stewardship. Probably, these findings could be explained by the fact the medical students who were in their clinical training years had more training and education about antimicrobial resistance and antimicrobial stewardship. Additionally, those students might have come across these issues during their on-site training in the hospitals. This might have contributed to their higher knowledge and attitudes towards antimicrobial resistance and antimicrobial stewardship. The findings reported in this study were consistent with those previously reported elsewhere among medical and healthcare students [17, 20, 21]. The findings of this study might be a call for educators and decision makers in medical schools to increase courses, educational, and training sessions relevant to antimicrobial resistance and antimicrobial stewardship early in the medical curricula.

The findings of this study showed that the vast majority of the medical students had positive attitudes towards integrating pharmacists, proper use of antibiotics, and antimicrobial stewardship. The findings reported in this study were consistent with those reported in other studies that were conducted elsewhere [14, 17]. The medical students also indicated that they have not received adequate education/training on the proper use of antibiotics. Probably, admitting lack of knowledge is a first step to accept future interventions that aim to bridge gaps of knowledge and increase awareness of the medical students about antimicrobial resistance and antimicrobial stewardship.

### Strengths and limitations

This is the first study in West Bank of Palestine to evaluate knowledge, perceptions, and attitudes of medical students towards antimicrobial resistance and antimicrobial stewardship. Second, a large number of medical students participated in this study. The medical students were of both sex, academic years, and had different demographic variables. Third, the tool used to assess knowledge, perceptions, and attitudes of medical students towards antimicrobial resistance and antimicrobial stewardship was previously used in previous studies.

On the other hand, the study was a cross-sectional. Cross-sectional lies at the bottom of the pyramid of hierarchy of evidence. Second, the questions used in this study were multiple choice (yes/no/I don’t know). This might have provided an opportunity for the students to guess and answer. Therefore, the answers might have been over-estimated.

#### Generalizability

Due to the varied backgrounds of students from just two medical schools in Palestine, the findings cannot be broadly applied, particularly considering the existence of approximately seven medical schools in the region. Thus, additional research is required to incorporate a more comprehensive representation of medical schools for a more inclusive analysis.

## Conclusion

The findings of our study indicate that medical students can benefit from more educational/training sessions about antimicrobial resistance and antimicrobial stewardship. The medical students are the future workforce of physicians in any healthcare system. Therefore, increasing knowledge of the medical students about and how to combat antimicrobial resistance can help save lives and improve patient outcomes. More studies are needed to find the best ways to increase knowledge, perceptions, and attitudes of medical students towards antimicrobial resistance and antimicrobial stewardship.

## Data Availability

The corresponding author will provide any information about this study upon reasonable request.
